# *In vitro* and *in vivo* assessment of hydroxypropyl cellulose as functional additive for enabling formulations containing itraconazole

**DOI:** 10.1016/j.ijpx.2021.100076

**Published:** 2021-03-17

**Authors:** Rafael D. Bachmaier, Marius Monschke, Thilo Faber, Anna K. Krome, Yann Pellequer, Edmont Stoyanov, Alf Lamprecht, Karl G. Wagner

**Affiliations:** a Department of Pharmaceutical Technology and Biopharmaceutics, University of Bonn, 53121 Bonn, Germany; b UFR Santé, Laboratoire de Pharmacie Galénique, 19, rue Ambroise Paré, 25000 Besancon, France; c Nisso Chemical Europe GmbH, Berliner Allee 42, 40212 Düsseldorf, Germany

**Keywords:** Amorphous solid dispersion, Hot-melt extrusion, HPC, HPMC, Biphasic dissolution, AcN, acetonitrile, API, active pharmaceutical ingredient, ASD, amorphous solid dispersion, AUC, area under the curve, BCS, biopharmaceutical classification system, DMSO, dimethyl sulfoxide, DSC, differential scanning calorimetry, FaSSIF, fasted state simulated intestinal fluid, GI, gastrointestinal, HME, hot-melt extrusion, HPC, hydroxypropyl cellulose, HPMC, hydroxypropyl methyl cellulose, ITZ, itraconazole, KTZ, ketoconazole, NCE, new chemical entity, OH-ITZ, hydroxy-itraconazole, PM, physical mixture, SD, spray-drying, T_G_, glass transition temperature, XRPD, x-ray powder diffraction

## Abstract

Using polymers as additives to formulate ternary amorphous solid dispersions (ASDs) has successfully been established to increase the bioavailability of poorly soluble drugs, when one polymer is not able to provide both, stabilizing the drug in the matrix and the supersaturated solution. Therefore, we investigated the influence of low-viscosity hydroxypropyl cellulose (HPC) polymers as an additive in HPMC based ternary ASD formulations made by hot-melt extrusion (HME) on the bioavailability of itraconazole (ITZ). The partitioning potential of the different HPC grades was screened in biphasic supersaturation assays. Solid-state analytics were performed using differential scanning calorimetry (DSC), X-ray powder diffraction (XRPD). The addition of HPCs, especially HPC-UL, resulted in a superior partitioned amount of ITZ in biphasic supersaturation assays. Moreover, the approach in using HPCs as an additive in HPMC based ASDs led to an increase in partitioned ITZ compared to Sporanox® in biorelevant biphasic dissolution studies. The results from the biphasic dissolution experiments correlated well with the *in vivo* studies, which revealed the highest oral bioavailability for the ternary ASD comprising HPC-UL and HPMC.

## Highlights

• Increased partitioning rate of itraconazole using low-viscosity HPC polymers.
• Enhanced bioavailability of itraconazole using HPC-UL as functional additive.
• Ternary amorphous solid dispersion with higher performance than Sporanox®.

## Graphical abstract

**Figure:**
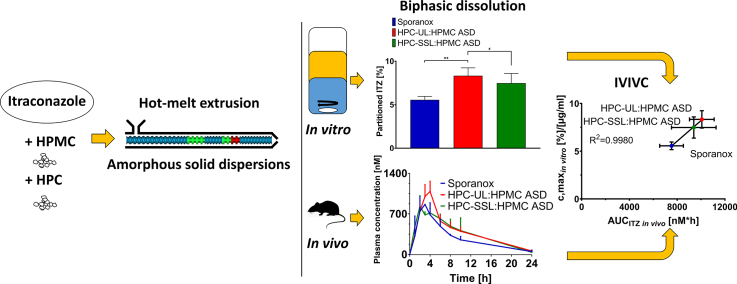

## 1 Introduction

Nowadays, the majority of new chemical entities (NCE) can be assigned to class II of the biopharmaceutics classification system (BCS) (Ku, 2008). Limited by being poorly water-soluble, BCS class II drugs are often accompanied by a low oral bioavailability (Lipinski, 2000). As BCS class II compounds exhibit a high permeability, the dissolution step within the gastrointestinal (GI) tract can be considered as decisive factor for the extent of the bioavailability (Horter and Dressman, 2001; Pouton, 2006). The generation of supersaturated drug solutions is known to facilitate the absorption rate within the GI passage (Stegemann et al., 2007). Promising formulation approaches therefore need to enable in a first step an improved kinetic solubility to generate a supersaturated solution and in a second step to maintain this supersaturated concentration for a prolonged period (Baghel et al., 2018a; Leuner, 2000; Monschke and Wagner, 2019; Vasconcelos et al., 2007). Various approaches in form of supersaturating drug delivery systems exist to enable an enhancement of dissolution profiles and oral bioavailability by the use of different functional excipients (Brouwers et al., 2009; Gao and Shi, 2012; Krome et al., 2020). It is important to differentiate the excipients functionality in regard to improve the solubility *e.g.*, as a matrix polymer of an amorphous solid dispersion (ASD) and the capacity of the excipient to maintain supersaturation.

Due to the fact that one polymer is not always capable to provide both, sufficient physical stabilization of the amorphous active pharmaceutical ingredient (API) and stabilization of the supersaturated solution upon dissolution, we previously proved the benefit to introduce an additional excipient to binary ASDs. The so called multicomponent ASDs such as ternary ASDs can thus further improve the dissolution performance and physical stability of poorly soluble drugs by rational selection of suitable polymer/excipient combinations (Prasad et al., 2014; Six et al., 2004; Yoo et al., 2009). The addition of a third component shall improve the physical stability and/or promote the dissolution by the inhibition of drug recrystallization (Baghel et al., 2018b).

**Fig. 1:**
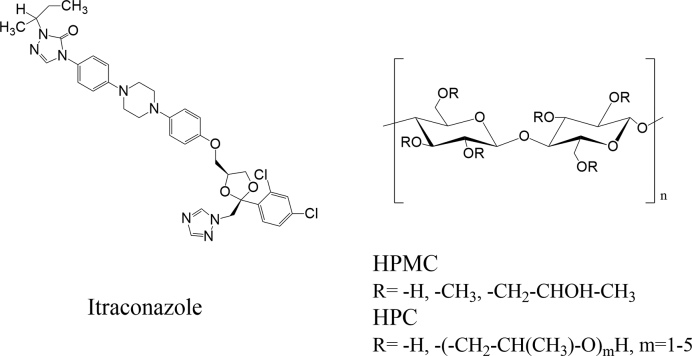
Chemical structures of ITZ, HPMC and HPC.

Hydroxypropyl cellulose (HPC) is a polymeric excipient which has been used as functional additive in ternary ASDs. HPC is a semisynthetic cellulose ether (Fig. 1) in which the hydroxyl groups of the cellulose backbone are additionally hydroxypropylated (Zhou et al., 2004). It is available in various chain lengths. In general, HPC is capable to stabilize supersaturated states of several APIs enabled by its precipitation inhibitory potential (Curatolo et al., 2009; Megrab et al., 1995). Due to its melt viscosity and thermal stability, HPC is also suitable as a polymeric carrier for the preparation of ASDs by means of hot-melt extrusion (HME) (Paradkar et al., 2009; Vasconcelos et al., 2007). However, the low glass transition temperature (*T*_g_) of low-viscosity HPCs (−25–0 °C) (Sarode et al., 2013) make those a less favorable matrix polymer for ASDs as inferior physical stability compared to HPMC, HPMCAS or copovidone can be expected. As an additive in ternary ASDs, HPC-SSL was successfully used in combination with HPMCAS as the main polymeric carrier to moderate the supersaturation of dipyridamole throughout the entire GI tract (Zecevic et al., 2014).

Itraconazole (ITZ) is an orally used triazole (Fig. 1) antifungal agent with a broad activity spectrum (De Beule and Van Gestel, 2001). The weak base (pK_a_ values of 2 and 3.7) can be categorized as a BCS class II drug, which is practically insoluble in water (~5 μg/ml at pH 1 and ~ 1 ng/ml at pH 7) and exhibits a high lipophilicity (logD value of 5.66 at pH 8.1) (Grant and Clissold, 1989; Ku, 2008; Peeters et al., 2002). ITZ is commercially available as Sporanox® capsules, a pellet-layered ASD formulation. Many different approaches have been investigated by using ITZ as a model substance to increase its oral bioavailability, such as nanocrystal-based, the combined use with mesoporous silica, solid dispersions or self-emulsifying systems (Sarnes et al., 2014; Van Speybroeck et al., 2010; Park et al., 2007).

Therefore, the aim of the present study was to investigate the influence of different low-viscosity HPC grades as an additive for ternary ASDs regarding the supersaturation potential and partitioning rate of ITZ (Ku, 2008; Peeters et al., 2002). The used HPC grades (HPC-UL, -SSL and -SL) differ in their molecular weight. In a first step, biphasic supersaturation assays (*i.e.*, solvent shift experiments) under biorelevant conditions were conducted in order to evaluate the ability of different HPC grades to promote the partitioning of ITZ into the organic phase as surrogate for absorption. Based on these tests, HME processed ternary ASDs containing HPMC and HPC as an additive were manufactured. These formulations were then investigated in biorelevant biphasic dissolution assays focusing on the ability of the tested formulations to promote the partitioning rate into the organic phase compared to the commercial product Sporanox®, which is often used as a benchmark formulation for *in vitro* as well as *in vivo* studies (Hong et al., 2006; Sarnes et al., 2014; Van Speybroeck et al., 2010). Biphasic dissolution tests have been successfully implemented in our group (Denninger et al., 2020; Frank et al., 2014; Monschke and Wagner, 2020). Moreover, with biphasic dissolution assays it is possible to predict *in vivo* performances of *e.g.* weak bases (Frank et al., 2014; Pestieau and Evrard, 2017). Therefore, the bioavailability of the ternary ASD formulations was investigated in *in vivo* studies in rats to assess the predictivity of our biphasic dissolution model and the potential of the enabling formulations using the marketed HPMC-based ASD Sporanox® as a benchmark.

## 2 Experimental

### 2.1 Materials

The model drug itraconazole (ITZ) was obtained from Sris Pharmaceuticals (Hyderabad, India). Hydroxy-itraconazole (OH-ITZ, 0.1 mg/ml methanol solution), the major active metabolite of ITZ in rats (Yoo et al., 2000) as well as in humans (Heykants et al., 1989), was obtained from Sigma-Aldrich (Taufkirchen, Germany). Ketoconazole (KTZ), the internal standard, was obtained from ThermoFisher (Kandel, Germany). Sporanox® 100 mg capsules were obtained from Janssen GmbH (Neuss, Germany). HPC-SL (M_W_ = 100,000 g/mol; 73.5% Hydroxypropoxy groups), -SSL (M_W_ = 40,000 g/mol; 73.7% Hydroxypropoxy groups) and -UL (M_W_ = 20,000 g/mol; 70.1% Hydroxypropoxy groups) were kindly donated by Nippon Soda Co., Ltd. (Tokyo, Japan). Affinisol® HME15LV (HPMC) was donated from Dow Chemical Company, (Bomlitz, Germany). LC-MS grade water was obtained from Bernd Kraft GmbH (Duisburg, Germany). LC-MS grade acetonitrile (AcN), potassium citrate monohydrate (> 99%) were obtained from VWR Chemicals GmbH (Darmstadt, Germany). Lecithin, sodium taurocholate, 1-decanol and potassium phosphate were obtained from ThermoFisher (Kandel, Germany). Sodium hydroxide was obtained from Honeywell Fluka (Seelze, Germany). 0.1 N HCl solutions were obtained from VWR Chemicals (Fontenay-sous-Bois, France). Dimethyl sulfoxide (DMSO, > 99.9%) was obtained from Fisher Scientific (Geel, Belgium).

### 2.2 Biorelevant biphasic supersaturation studies

In order to investigate the partitioning rate of ITZ in presence of the different HPC grades, biphasic supersaturation studies were performed using the BiPHa+ apparatus developed by Denninger et al. (Denninger et al., 2020). The apparatus mainly consists of four cylindrical vessels (one blank and three sample vessels) set into a water bath with a temperature of 37 ± 0.5 °C. These vessels were each filled with 50.0 ml of the aqueous phase. For generation of the aqueous phase, each HPC grade was previously dissolved in 0.1 N HCl solution to obtain polymer concentrations of 0.250% (w/v), 0.125% (w/v) and 0.063% (w/v). Furthermore, triangular magnetic stirrer were put into the vessels at 170 rpm to gain sufficient hydrodynamics. The biorelevant biphasic supersaturation studies were divided into three pH stages. The steps were: 15 min at pH 1.0, 45 min at pH 5.5 and 90 min at pH 6.8. The pH shifts were conducted by the use of a concentrated citrate-phosphate buffer. The pH was monitored during the whole experiments. An ITZ stock solution (10 mg/ml) in DMSO was added to 50 ml of each prepared vessel by targeting a concentration of 0.1 mg/ml of ITZ. Immediately prior the pH adjustment to 5.5, a stock solution of sodium taurocholate and lecithin was added to the aqueous phase to obtain a fasted state simulated intestinal fluid (FaSSIF)-V2 like biorelevant medium (Denninger et al., 2020). Subsequently, 50.0 ml of 1-decanol were added to create the absorptive sink medium. Both, the adjustment of the pH and addition of the absorptive sink, were performed by a fully automated liquid dispenser. For the quantification of the ITZ concentration in the aqueous phase and the absorptive sink, an Agilent 8454 diode array UV spectrophotometer was used.

The partitioned amount of ITZ in the organic phase at the last time point was statistically compared by using the one-way analysis of variance (ANOVA) and a *post hoc* Tukey's multiple comparison test.

### 2.3 Preparation of amorphous solid dispersions *via* hot-melt extrusion (HME)

A co-rotating 12 mm twin-screw extruder ZE 12 from Three-Tec GmbH (Seon, Switzerland) with a functional length of 25:1 L/D and a 2 mm die was used for the preparation of the ternary ASDs. The composition of the formulations and the extruder conditions and properties are described in Table 1. The feed rate was set to 2 g/min. The composition of the ternary ASDs was determined based on preliminary studies. HPC alone is not suitable to create single phased systems containing ITZ. However, HPMC (shown on Sporanox®) is suitable to incorporate ITZ within its polymeric matrix. Thus, different mixtures of HPC and HPMC were prepared with ITZ in order to make transparent films *via* film casting. The ratio of 1:2 (HPC:HPMC) was suitable to produce transparent films, indicating a single phased ASD. The extrusion temperature was chosen based on the HPMC supplier's recommendation. The screw speed and feed rate was adopted according to previous publications (Monschke et al., 2020; Monschke and Wagner, 2020; Monschke and Wagner, 2019).

**Table 1:** Composition of the formulations, extrusion conditions and resulting properties.

Name	ITZ:HPC:HPMC	Temperature [°C]	Screw speed [rpm]	Torque [Nm]
ITZ:HPC-UL:HPMC 1:3:6 HME	1:3:6	100/150/150/150/150	100	5.0
ITZ:HPC-SSL:HPMC 1:3:6 HME	1:3:6	100/150/150/150/150	100	6.0

### 2.4 X-ray powder diffraction (XRPD)

XRPD was performed in reflection mode (X'Pert MRD Pro, PANalyical, Almelo, the Netherlands) with an X'Celerator detector and nickel filtered CuKα1 radiation at 45 kV and 40 mA. The scanning range was set in a 2θ range from 4° to 45° with a step size of 0.017°. Data were evaluated with X'Pert High Score version 2.2.

### 2.5 Differential scanning calorimetry (DSC)

DSC measurements were performed using a Mettler-Toledo DSC 2 (Gießen, Germany) equipped with a nitrogen cooling system and nitrogen as purge gas (30 ml/min). Both extruded ASDs were weighed (8–15 mg) into aluminum pans with a pierced lid. The determination of the glass transition temperatures was carried out in TOPEM-mode (DSC measurements with multi-frequency temperature modulation) with a constantly heating rate of 2 K/min using a scanning range from 2 °C to 180 °C.

### 2.6 Biorelevant biphasic dissolution studies

Sporanox® and the ASDs of ITZ with Affinisol® HME15LV (HPMC) and HPC-UL/HPC-SSL (ITZ:HPC-UL/-SSL:HPMC 1:3:6) were tested in biphasic dissolution studies. The same apparatus and conditions were used as for the biphasic supersaturation studies (2.2.). Instead of polymeric solutions, pure 0.1 N HCl solution was used for the starting medium of pH 1.0. The duration time was set to 60 min at pH 1.0, 45 min at pH 5.5 and 90 min at pH 6.8.

An amount equivalent to 5 mg of ITZ was used for the studies. In case of Sporanox®, the capsules were opened and an amount equivalent to 5 mg ITZ was withdrawn and used for biorelevant biphasic dissolution. The partitioned amount of ITZ in the absorptive sink at the last time point was statistically compared by using the one-way analysis of variance (ANOVA) and a *post hoc* Tukey's multiple comparison test.

### 2.7 Stability studies

To determine the physical stability of the hot-melt extruded formulations regarding recrystallization of ITZ, milled extrudates in capped glass vials were stored at 25 °C, closed with desiccant for 6 months and analyzed by XRPD (as described in 2.4).

### 2.8 Animal studies

#### 2.8.1 Animals

For *in vivo* studies, the three formulations Sporanox®, ITZ:HPC-UL:HPMC 1:3:6 HME and ITZ:HPC-SSL:HPMC 1:3:6 HME were tested each on three male Sprague-Dawley rats (280–350 g, approx. 9 weeks of age). All animal experiments were conducted according to the European Union Directive 2010/63/EU. Those studies were performed at the University of Bourgogne Franche-Comté (Besancon, France) in compliance with the French legislation and European Community Guidelines on animal experimentation under the authorization number “Project Exp An N2 EA4267 2015-2020”, accepted by the ethical committee CEBEA 58. The animals were acclimatized for one week and fasted for 12 h before the application with constant access to water. After application of the formulations (*t* = 0), food was rearranged.

#### 2.8.2 Dosing and blood sampling

Each formulation was dosed orally equal to 5 mg of ITZ, as either Sporanox® pellets or ASDs, dispersed in 500 μl water. All formulations were dosed *via* an oral gavage. Blood samples of 300 μl were collected by individual venipunctures of the lateral tail vein after 1, 2, 3, 4, 6, 8, 10 and 24 h after and 0.5 h before the formulation application in lithium heparinized centrifugation tubes (25 I.U./ml, 1.3 ml, Sarstedt AG & Co. KG, Nümbruch, Germany). To obtain plasma, the blood samples were centrifuged (3600*g*, 4 °C, 10 min) using a ThermoFischer Heraeus Multifuge X1 (Thermo Electron LED GmbH, Osterode, Germany) and kept frozen at −70 °C until further preparation and analysis by LC-MS.

#### 2.8.3 Sample preparation

20 μl of plasma was added into an Eppendorf tube. After addition of 20 μl of an internal standard solution (containing 1200 ng/ml KTZ in AcN) and 40 μl of ice-cold AcN, the Eppendorf tubes were vortexed for 60 s and centrifuged (25 min, 11,000 rpm, 4 °C) using a ThermoFischer Heraeus Fresco 17 centrifuge (Thermo Electron LED GmbH, Osterode, Germany). The supernatant was then transferred into LC vials (Waters Screw Neck Total Recovery Vials, Eschborn, Germany) and analyzed by LC-MS as described in the next section. The same procedure was performed for the calibration. A certain amount of calibration stock solution was diluted by centrifuged plasma (from purchased raw rat blood) 20-fold and underwent the same plasma extraction procedure as the collected plasma samples.

#### 2.8.4 LC-MS studies and data analysis

The quantification of ITZ and OH-ITZ (the major active metabolite of ITZ in humans (Heykants et al., 1989) as well as in rats (Yoo et al., 2000)) was performed for all plasma samples. For the bioanalytics, a Waters e2695 Separation Module coupled to a Waters ACQ-QDA Detector was used. MS-measurements were performed with a capillary voltage of 0.8 kV and a cone voltage of 15 V. A Waters XBridge® Shield RP18 column (3.5 μm, 2.1 mm × 100 mm, 130 Å) was installed and the column temperature set to 30 °C. Non-isocratic conditions were applied to measure the ITZ and OH-ITZ plasma concentrations precisely. The compositions of the mobile phases and the non-isocratic conditions are described in Table 2, Table 3. For detection, the positive ion electrospray ionization mode was used. Quantification was performed with an internal standard method using KTZ as the internal standard.

**Table 2:** Composition of the mobile phases used for the LC-MS measurements.

Excipient	Mobile phase A		Mobile phase B
Water	19	:	1
Acetonitrile	1	:	19
Ammonium acetate	0.039% (w/v)		
Glacial acetic acid	+ 0.1 ml

**Table 3:** Non-isocratic conditions of the LC-MS measurements.

Time [min]	Mobile phase A [%]	Mobile phase B [%]	Flow rate [ml/min]
0	80	20	0.3
20	20	80	0.3
30	20	80	0.3
32	80	20	0.3
40	80	20	0.3

#### 2.8.5 Data analysis

The maximum plasma concentration (c_max_) and the associated time t_max_ were determined directly from the plasma concentration profiles. The determination of the area under the curve (AUC) of the plasma concentration profiles were estimated by trapezoidal integration to the last sampling point (24 h). The evaluation of the bioavailability was performed by using the sum of the AUCs of ITZ and OH-ITZ (AUC_sum_). The statistical comparison of c_max_ and AUC_ITZ_ were accomplished with a one-way analysis of variance (ANOVA) and *post hoc* Tukey's multiple comparison test. *P* values of <0.05 were considered as statistically significant.

## 3 Results and discussion

### 3.1 Biphasic supersaturation

**Fig. 2:**
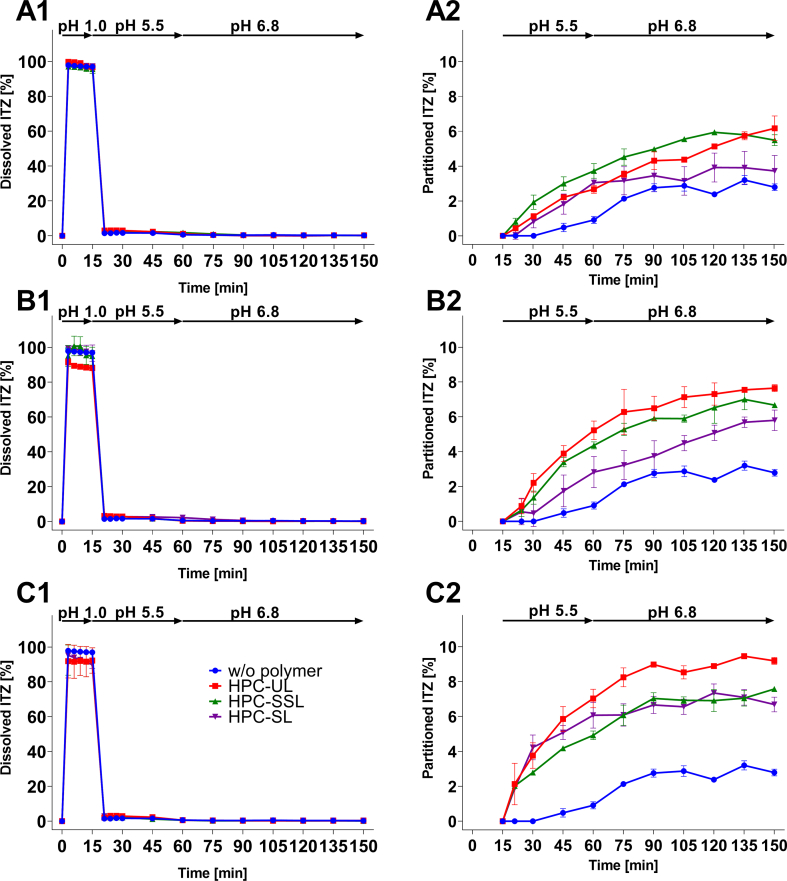
Biphasic supersaturation studies of ITZ in colloidal solutions of HPC of various grades and concentrations of 0.063% (w/v) (aqueous: A1; organic: A2), 0.125% (w/v) (aqueous: B1; organic: B2) and 0.250% (w/v) (aqueous: C1; organic: C2); target concentration: 0.1 mg/ml = 100%; *n* = 3, mean ± SD.

**Fig. 3:**
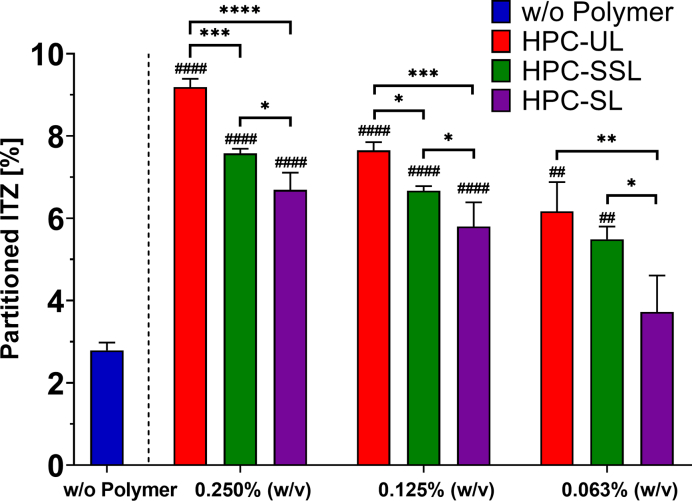
Comparison of the partitioned amount of ITZ into the organic solvent layer after 150 min of the biphasic supersaturation assays; target concentration: 0.1 mg/ml = 100%, n = 3, mean ± SD. * *p* < 0.05; ** *p* < 0.01; *** *p* < 0.001; **** *p* < 0.0001. Statistical significance in reference to ITZ w/o polymer: # p < 0.05; ## p < 0.01; #### p < 0.0001.

The results of the biphasic supersaturation assays are provided in Fig. 2. At pH 1.0, ITZ completely remained in solution after it was added in form of DMSO stock solutions, independent on the presence or absence of an HPC polymer. Continued with the first pH shift and the addition of the FaSSIF-V2 concentrate, ITZ started to precipitate in all cases resulting in a cloudy aqueous suspension with visible precipitates related to the change of ITZ from a positively charged species into an uncharged (Peeters et al., 2002). For the period between 45 and 60 min, the presence of the different HPC polymers led to a concentration of approx. 1–3% ITZ in the aqueous phase. After that, the dissolved amount of ITZ in the aqueous phase dropped to approx. 0.8% and the ITZ partitioning rate into the absorption sink was reduced independent on the tested polymer and the tested polymer concentration. Despite the fact, that there was no significant difference among all tested samples regarding the ITZ concentration in the aqueous phase after the pH shift, the partitioned amount of ITZ into the organic solvent layer clearly differed. By increasing the concentration of each HPC polymer, the partitioned amount of ITZ was increased and the difference between the HPC grades was less pronounced. The presence of all tested HPC grades, however, significantly increased the partitioned amount of ITZ into the absorption sink regardless of the polymer concentration compared to pure ITZ. Only HPC-SL at a concentration of 0.063% (w/v) did not result in a significant higher concentration compared to the pure ITZ. The presence of HPC-UL resulted in the fastest partitioning rate and thus for all concentrations in the highest partitioned amount of ITZ, respectively (0.063% (w/v): 6.17% ITZ; 0.125% (w/v): 7.65% ITZ; 0.250% (w/v): 9.19% ITZ, after 150 min, Fig. 3). HPC-UL resulted at all concentrations except for 0.063% (w/v) in a significant higher distribution of ITZ than HPC-SSL. At polymer concentrations of at least 0.125% (w/v) a rank order of UL > SSL > SL in terms of ITZ partitioning was observed (Fig. 3). The lower molecular weight HPC polymer HPC-UL exhibited the greatest effect on promoting the partitioning of ITZ. In all cases, the partitioning rate of ITZ into the absorption sink decreased after approx. 60 min after the pH shift, likely due to the further concentration reduction caused by the second pH-shift to pH 6.8.

HPC polymers have been successfully tested in the past in stabilizing the supersaturation of poorly soluble drugs with different physicochemical properties *e.g.* ibuprofen, carbamazepine, phenytoin and chlorphenamine (Sarode et al., 2013; Terebetski et al., 2014). Furthermore, it was also shown, that an HPC-SSL based formulation promoted the partitioning of felodipine in the n-octanol layer during biphasic dissolution assays compared to the neat API (Sarode et al., 2014). The ITZ concentrations in the aqueous phase were too low to distinguish the effect of the HPC polymers with regard to supersaturation, whereas significant differences in the organic solvent layer might be related to enhanced re-dissolution kinetics of the precipitated particles (Frank et al., 2014). This phenomenon was also reported during the biphasic dissolution studies of ritonavir under biorelevant conditions (Denninger et al., 2020).

### 3.2 Solid-state analytics

**Fig. 4:**
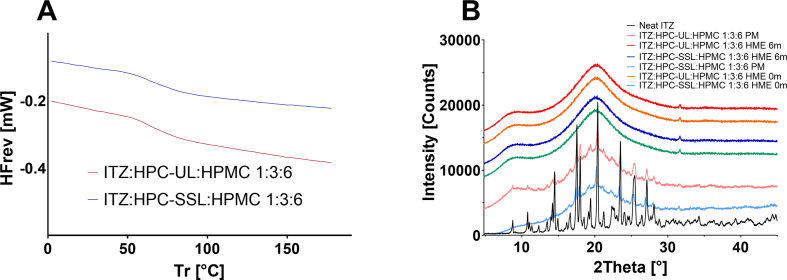
Solid-state analytics of the studied formulations. XRPD of (A) DSC of the hot-melt extruded ITZ:HPMC:HPC-UL/-SSL 1:6:3 ASDs after processing and (B) the hot-melt extruded ITZ:HPMC:HPC-UL/-SSL 1:6:3 HME ternary ASDs after processing and after 6 months under stability conditions in comparison to their physical mixtures (PM) and neat ITZ.

Fig. 4 shows the solid-state analytics of all formulations. Main peaks of ITZ are clearly shown at 14.5° 2θ, 17.5° 2θ, 18.0° 2θ, 20.4° 2θ. 23.5° 2θ, 25.5° 2θ and 27.1° 2θ for the neat substance as well as the physical mixtures. The comparison between the HME processed components and their physical mixtures showed, that ITZ was amorphous within the solid dispersions by showing a diffuse halo with no Bragg peaks of ITZ in the XRPD diffractograms. After 6 months under stability conditions, the solid-state of the ASDs did not change, indicating an acceptable physical stability. Minor peaks in the diffractograms of the ternary ASDs shown at approx. 32° 2θ can be explained by the presence of residual sodium chloride during the manufacturing process of Affinisol® HME15LV. The DSC thermograms revealed a glass transition temperature at 70.5 °C for ITZ:HPMC:HPC-UL 1:6:3 ASD and at 72.7 °C for the ITZ:HPMC:HPC-SSL 1:6:3 ASD. The presence of one single glass transition temperature and the absence of a melting endotherm indicated single phased ASDs.

### 3.3 Biphasic dissolution studies

**Fig. 5:**
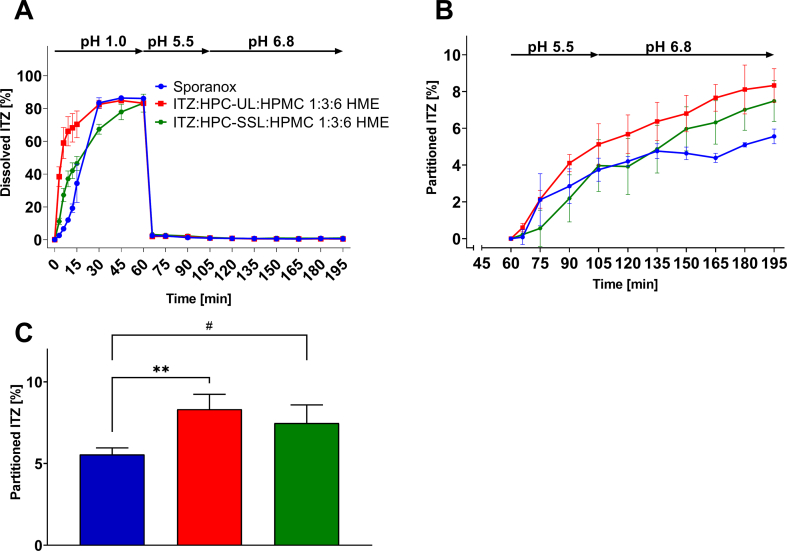
Biphasic dissolution profiles of Sporanox® and the self-manufactured ternary ASDs (aqueous: A; organic B); target concentration: 0.1 mg/ml = 100%; n = 3, mean ± SD and (C) comparison of the into the organic solvent layer partitioned ITZ after 195 min of the biphasic dissolution assays of Sporanox® and both hot-melt extruded ASDs; target concentration: 0.1 mg/ml = 100%, n = 3, mean ± SD. # *p* < 0.1; ** *p* < 0.01.

Fig. 5 shows the *in vitro* biphasic dissolution tests of the ternary ASDs in comparison to Sporanox®. All ternary ASDs dissolved completely within the first 60 min under acidic conditions (Fig. 5A). The solid-state analytics revealed single-phase systems which proved that ITZ was molecularly dispersed within the polymeric carrier. Hence, the dissolution rate of ITZ was controlled by the dissolution rate of HPMC and HPC-UL or -SSL. It is worth mentioning that ITZ:HPC-UL:HPMC 1:3:6 HME dissolved faster than the SSL formulation as a lower molecular weight increased the dissolution kinetics of the ASD polymer (Sarode et al., 2013).

Both ternary ASDs exhibited a faster dissolution rate compared to Sporanox® within the first 15 min. Due to the fact, that the formulations were already dissolved before the pH shifted and precipitated prior the addition of 1-decanol, the dissolution rates of the formulations did not have an influence on the partitioning rate of ITZ into the organic phase. However, the presence of the HPC polymers affected the partitioned amount of ITZ, despite the fact, that there were no significant differences in the aqueous phase. Sporanox® partitioned approximately 5.5% of ITZ into the organic layer after 195 min. In comparison, both ternary ASDs resulted in significant higher partitioned ITZ into the organic solvent layer. The ITZ:HPC-UL:HPMC ASD partitioned 8.33% (*p* < 0.05) compared to Sporanox® (5.56%) into the 1-decanol phase (Fig. 5, B + C), while the dissolution test of the ASDs containing HPC-SSL resulted in a partitioned amount of 7.48% ITZ (*p* < 0.1 compared to Sporanox®. It was shown, that HPMC is capable of stabilizing supersaturated ITZ solutions (Verreck et al., 2003). An addition of different HPC grades, however, showed no difference in the aqueous phase within the biphasic supersaturation assays as a consequence of the extremely low solubility of ITZ at pH values above its pK_a_. However, the addition of HPC-SSL and especially HPC-UL promoted the partitioning rate of ITZ into the organic solvent layer, even in combination with HPMC. This might be related to faster re-dissolution kinetics of the precipitate, facilitated by the presence of HPC (Frank et al., 2014; Xie et al., 2017). Denninger et al. (2020) showed that the transition into the organic phase could be of significant difference despite the fact that the aqueous concentration was on the same level. They revealed that the difference in partitioning rate was related to the re-dissolution kinetics of the precipitate.

### 3.4 Animal Studies

**Fig. 6:**
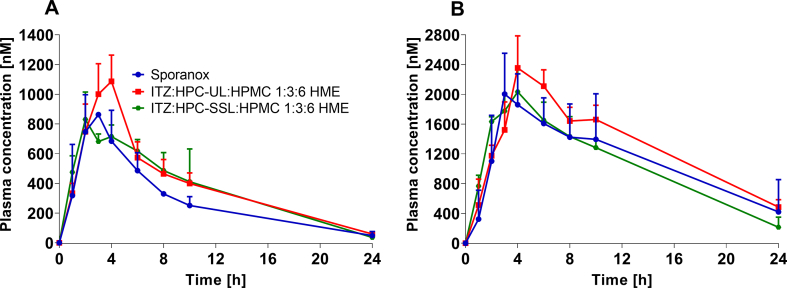
Plasma ITZ (A) and OH-ITZ (B) concentration profiles of Sporanox® and both hot-melt extrusion-processed ITZ:HPMC:HPC-UL or -SSL 1:6:3 HME after oral administration equal to 5 mg ITZ; n = 3; mean ± SD.

For the *in vivo* experiments, both ternary ASDs were tested in comparison to Sporanox®. The biopharmaceutical performance of all the formulations exhibited the same rank order *in vitro* as well as *in vivo*. The only exception was observed for ITZ:HPMC-SSL:HPMC 1:3:6 HME, which was almost at the same level as Sporanox® *in vivo*. A summary of the plasma concentration profiles and the PK parameters of all formulations that were evaluated *in vivo* is provided in Fig. 6 and Table 4. The ITZ:HPC-UL:HPMC 1:3:6 HME ASD exhibited a significantly higher AUC_ITZ_ than Sporanox® (*p* < 0.1). Considering the c_max_ values, between all formulations, a significant difference was present, except for the c_max_ values between Sporanox® and ITZ:HPC-SSL:HPMC 1:3:6 HME. The use of HPC-SSL as an additive did not increase the bioavailability of ITZ *in vivo* compared to Sporanox®. ITZ:HPC-UL:HPMC 1:3:6 HME stood out by showing a higher AUC_sum_ than Sporanox® (14% higher compared to Sporanox®).

**Table 4:** Summarized results of the animal studies (n = 3). Average and standard deviation are given for AUC and c_max_ values. Median and range for t_max_.

Formulation	AUC _ITZ_ [nM*h]	c_max ITZ_ [nM]	t_max ITZ_ [h]	AUC_OH-ITZ_ [nM*h]	c_max OH-ITZ_ [nM]	t_max OH-ITZ_ [h]	AUC_sum_ [nM*h]	AUC_sum_ relative to Sporanox® [%]
Sporanox®	7529 ± 992	891 ± 46	3 (2–3)	29,695 ± 2454	2204.3 ± 381.2	3 (3–4)	37,224 ± 1088	100
ITZ:HPC-UL:HPMC 1:3:6 HME	10,462 ± 1033	1211 ± 35	3 (3–4)	31,953 ± 3174	2387.3 ± 374.3	4 (3–6)	42,415 ± 2776	114
ITZ:HPC-SSL:HPMC 1:3:6 HME	9391 ± 1865	889 ± 95	2 (2–4)	26,442 ± 5103	2149.3 ± 170.5	4 (3–4)	35,832 ± 6232	96

**Fig. 7:**
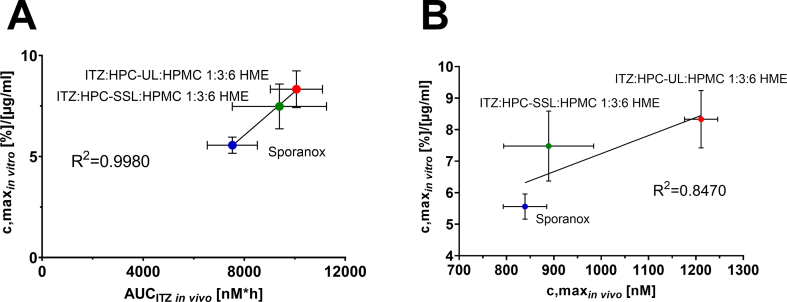
*In vitro-in vivo-*relationship between the maximum concentration after 195 min in the organic solvent layer of the *in vitro* biphasic dissolution studies and either (A) the AUC of ITZ *in vivo* or (B) the maximum plasma concentration measured *in vivo* for all tested formulations.

It has been reported for Sporanox®, that it may prolong and delay the ITZ retention time in the stomach as a consequence of the particle size of the pellets (Sarnes et al., 2014; Sarnes et al., 2013). In the small intestine, which represents the main absorption domain for ITZ (Six et al., 2004), the solubility of ITZ is lower than in the stomach and supersaturated solutions are also less stable and precipitate, caused by the higher pH (Sharma et al., 2009). In case of Sporanox®, the stomach acted thus as a reservoir from which stabilized ITZ could be delivered to the small intestinal where ITZ could gradually be absorbed (Matteucci et al., 2009), which might explain a slightly better performance of Sporanox® *in vivo* in comparison to the *in vitro* results. The rank order of the biopharmaceutical performance *in vivo* aligned well with the *in vitro* dissolution studies of the tested formulations under biorelevant conditions. The almost similar profiles of ITZ:HPMC:HPC-SSL 1:3:6 HME and Sporanox® *in vivo*, might be related to an almost comparable partitioning profile of both during the first 120 min *in vitro*. Fig. 7 depicts the *in vitro-in vivo* relationship of the tested formulations. The final concentration of the biphasic dissolution tests after 195 min correlated well with the evaluated AUC of ITZ *in vivo* by having a regression coefficient of 0.9980, whereby the performance of Sporanox® was slightly underestimated, because biphasic dissolution tests could not reflect the enhanced transit time of the Sporanox® pellets.

The comparability of the *in vivo* and the *in vitro* results showed that the biphasic dissolution model in this study was able to show a relationship between the partitioned amount *in vitro* and the absorbed ITZ *in vivo*. Nevertheless, it is not uncommon that there exists deviations of *in vivo* and *in vitro* results of ITZ formulations (Do et al., 2011), also due to the high variability of ITZ *in vivo* (Prentice and Glasmacher, 2005).

## 4 Conclusion

The present study showed that the addition of low-viscosity HPC polymers, especially HPC-UL, could be a useful functional additive for enabling formulations containing ITZ. The supersaturation assays showed the potential of the HPC polymers to promote the ITZ partition into an organic solvent layer which might be caused by an increased re-dissolution. As predicted by the biphasic dissolution experiments, the ternary ASD containing HPMC and HPC-UL resulted in the highest oral bioavailability compared to all other formulations including Sporanox®.

## Declaration of Competing Interest

This work was funded by Nisso Chemical Europe GmbH. University of Bonn and Nisso Chemical Europe GmbH participated in study design, research, interpretation of data, writing, data collection, analysis, reviewing, and approving the publication. Edmont Stoyanov is an employee of Nisso Chemical Europe GmbH and does not own stocks on Nippon Soda Ltd. Rafael Bachmaier, Marius Monschke, Anna K. Krome and Thilo Faber are PhD students and Karl G. Wagner and Alf Lamprecht are professors at the University of Bonn. Yann Pellequer is an employee of the University of Franche-Comté in Besancon. They have no additional conflicts of interest to report.
